# The Potential of Cannabidiol as a Treatment for Psychosis and Addiction: Who Benefits Most? A Systematic Review

**DOI:** 10.3390/jcm8071058

**Published:** 2019-07-19

**Authors:** Albert Batalla, Hella Janssen, Shiral S. Gangadin, Matthijs G. Bossong

**Affiliations:** 1 Department of Psychiatry, UMC Utrecht Brain Center, University Medical Center Utrecht, 3508 GA Utrecht, The Netherlands; 2 Section of Neuropsychiatry, Department of Biomedical Sciences of Cells and Systems, University Medical Center Groningen, 9713 AV Groningen, The Netherlands

**Keywords:** cannabidiol, CBD, cannabis, psychosis, schizophrenia, substance use disorders, addiction

## Abstract

The endogenous cannabinoid (eCB) system plays an important role in the pathophysiology of both psychotic disorders and substance use disorders (SUDs). The non-psychoactive cannabinoid compound, cannabidiol (CBD) is a highly promising tool in the treatment of both disorders. Here we review human clinical studies that investigated the efficacy of CBD treatment for schizophrenia, substance use disorders, and their comorbidity. In particular, we examined possible profiles of patients who may benefit the most from CBD treatment. CBD, either as monotherapy or added to regular antipsychotic medication, improved symptoms in patients with schizophrenia, with particularly promising effects in the early stages of illness. A potential biomarker is the level of anandamide in blood. CBD and THC mixtures showed positive effects in reducing short-term withdrawal and craving in cannabis use disorders. Studies on schizophrenia and comorbid substance use are lacking. Future studies should focus on the effects of CBD on psychotic disorders in different stages of illness, together with the effects on comorbid substance use. These studies should use standardized measures to assess cannabis use. In addition, future efforts should be taken to study the relationship between the eCB system, GABA/glutamate, and the immune system to reveal the underlying neurobiology of the effects of CBD.

## 1. Introduction

Schizophrenia is a complex mental disorder, which has a profound impact on patients. The burden of schizophrenia is explained by the early onset, often in early adulthood or late adolescence, its chronic course, and its relatively high prevalence [1]. The symptomatology is highly heterogeneous and often overlaps with comorbid disorders, such as affective or substance use disorders [2,3]. Psychotic symptoms are grouped into three dimensions: Positive symptoms (e.g., delusions, hallucinations), negative symptoms (e.g., blunted affect, anhedonia), and cognitive symptoms (e.g., attention, memory, executive functioning; see for reviews [4,5,6]). Different combinations of symptoms and comorbidity lead to different clinical profiles and treatment needs. However, the pharmacological treatment of schizophrenia is mainly based on dopamine blockade, the effect of which is limited to the positive symptoms [7]. Moreover, two-thirds of the patients experience a suboptimal response with dopaminergic treatment [8], and these results are even worse when comorbid substance use disorders (SUDs) are present [9]. Therefore, there is an urgent need for alternative and more effective pharmacological interventions aimed to reduce the burden of complex and overlapping symptom profiles. 

One of these interventions may involve the endocannabinoid (eCB) system, which is a promising new pharmacological target in this respect. The eCB system consists of at least two types of receptors and their endogenous ligands (i.e., endocannabinoids; [10,11]). The cannabinoid receptors are predominantly present in the central nervous system, in particular, in several limbic and cortical brain structures [12]. The eCB system is a retrograde messenger system that regulates both excitatory glutamate and inhibitory GABA neurotransmission according to an ‘on-demand’ principle: Endocannabinoids are released when and where they are needed [10,11,13]. This endocannabinoid-mediated regulation of synaptic transmission is a widespread phenomenon in the brain and is thought to play an important role in higher brain functions, such as cognition, motor function, and processing of sensory input, reward, and emotions [14,15,16,17]. eCB receptors are also present on immune cells in the central nervous system (i.e., microglia), which suggests their involvement in processes such as cytokine release, immune suppression, and induction of both cell migration and apoptosis [18,19].

The role of the eCB system in the pathophysiology of schizophrenia has been suggested in an accumulating amount of evidence [20,21]. First, epidemiological studies suggest that cannabis use increases the risk for developing schizophrenia [22] and lowers the age of onset of the disorder [23,24]. This risk increases with a higher frequency of cannabis use (e.g., daily use), and with the consumption of more potent cannabis (i.e., a higher amount of Δ9-tetrahydrocannabinol; THC) [22,25,26,27]. Second, modulation of the eCB system by the administration of THC (i.e., the main psychoactive component in cannabis) to healthy volunteers showed that THC can induce positive psychotic symptoms, effects that resemble negative symptoms (e.g., blunted affect, lack of spontaneity) and deficits in cognition (reviewed in [28]). Importantly, in schizophrenia patients, enhanced levels of endocannabinoids were demonstrated in cerebrospinal fluid and blood [29,30,31], and increased CB receptor density and availability were shown in the brain [32,33]. 

In addition to its role in schizophrenia, there is overwhelming evidence that the eCB system is implicated in the pathophysiology of addiction, in particular in processes such as drug-seeking behaviour, reward, withdrawal, and relapse (see for reviews [34,35,36,37]). For example, animal studies have shown that addictive properties reflected in behaviours such as self-administration or conditioned place preference of opiates, nicotine, and alcohol are absent or attenuated in cannabinoid CB1-receptor knockout mice and after administration of CB1 antagonists [35]. In addition, whereas the drug seeking behaviour of drugs of abuse was blocked with CB1 antagonists, it was reinstated after the administration of CB1 agonists [34,36]. Finally, endocannabinoid concentrations are affected by active drug seeking behaviour and eCB signalling seems to modulate the rewarding effects of addictive drugs [38]. 

SUDs and psychotic disorders such as schizophrenia co-occur frequently. Prevalence rates of any SUD (excluding nicotine and caffeine) in patients with schizophrenia are up to 45% [39,40], with the most frequently used substances being cannabis and alcohol. Considering nicotine use disorders, the prevalence rates rise up to 60%–90% [40]. Persistent use of licit or illicit drugs has been associated with adverse consequences in the overall course of psychotic disorders, and increased morbidity and mortality [40]. In addition, SUDs are also related to poor medication adherence, increasing the risk of relapse [39]. For example, in patients with schizophrenia, cannabis use has been related to higher relapse rates, increased severity of symptoms, and poor outcome [41,42,43,44,45]. Despite the high co-occurring rates, patients with comorbid SUDs and psychotic disorders are often excluded from clinical trials, which limits the generalization of results and ignores the potential (positive or negative) effects of the intervention on substance use.

While THC can trigger both schizophrenia and SUD and worsen the course of both disorders, the non-psychoactive cannabinoid compound cannabidiol (CBD) may have opposite or even beneficial effects. For example, CBD may have the ability to counteract psychotic symptoms and cognitive impairment associated with cannabis use as well as with acute THC administration [46,47]. In addition, CBD may lower the risk for developing psychosis that is related to cannabis use [48]. These effects are possibly mediated by the opposite effects of CBD and THC on brain activity patterns in key regions implicated in the pathophysiology of schizophrenia, such as the striatum, hippocampus, and prefrontal cortex [28]. Therefore, CBD displays a highly favourable profile for development as a new antipsychotic agent [48]. Similarly, CBD may serve as a treatment for SUDs, since evidence from preclinical studies suggests that CBD reduces negative withdrawal effects, motivation for self-administration, and reinstatement of drug use [37]. As a result, CBD-containing compounds are increasingly being investigated in the context of substance abuse in humans as well. 

The eCB system appears an interesting target for schizophrenia, SUDs, and their comorbidity, due to the implication of the eCB system in their pathophysiology and the beneficial effects of CBD in both disorders. However, one may expect that CBD treatment may be most effective in a subgroup of patients, for example patients who show alterations in the eCB system or have a specific symptom profile. CBD may restore an imbalance in the eCB system, which may result in clinical improvement. Although previous excellent reviews (e.g., [37,48,49]) described the potential of CBD as a treatment for psychosis and SUD, this review provides a detailed and up-to-date systematic literature overview of clinical studies that investigated the efficacy of CBD treatment for schizophrenia and/or SUD. In addition, this review examined whether there are specific subgroup of patients with schizophrenia, SUD, or both that may benefit the most from CBD treatment. 

## 2. Experimental Section

Clinical trials and case reports published up to February 2019, which described the effects of CBD on the symptomatology of psychotic disorders (i.e., schizophrenia and related disorders), SUD, or both were included. Reviews, non-English articles, pre-clinical or animal studies, studies that investigate CBD tolerability and pharmacokinetics or compare the acute effects of CBD with THC, and articles describing psychiatric or neurologic disorders other than psychotic disorders and SUD were excluded.

A literature search was conducted in the PubMed database. The following two searches were used: (1) “(((cannabidiol [MeSH Terms]) OR CBD[Text Word])) AND ((((((Substance-Related Disorders[MeSH Terms]) OR addiction[Text Word]) OR addictive behavior[Text Word]) OR drug abuse[Text Word])) OR drug dependence[Text Word])”, (2) “(((((((Schizophrenia Spectrum and Other Psychotic Disorders[MeSH Terms])) OR schizophrenia[Text Word]) OR schizophrenic[Text Word]) OR psychosis[Text Word]) OR psychotic[Text Word])) AND ((cannabidiol[MeSH Terms]) OR CBD[Text Word])”.

## 3. Results

**Figure 1:**
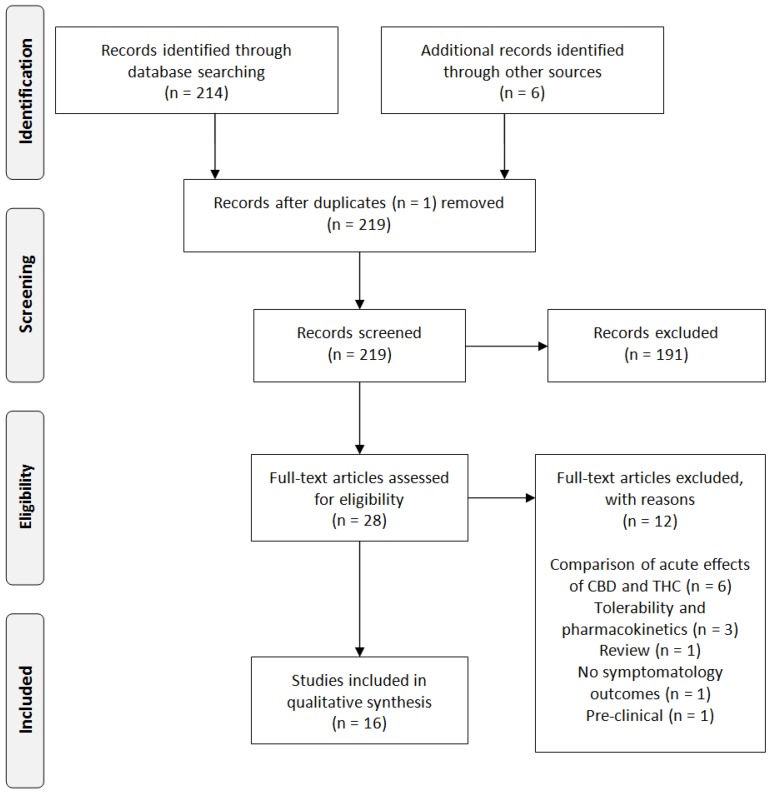
Study inclusion process. CBD: Cannabidiol; THC: Δ9-Tetrahydrocannabinol.

The searches resulted in 214 articles, which included one duplicate (Figure 1). The articles were screened by two authors independently, according to the PRISMA guidelines [50]. After full-text screening, ten articles from the systematic search were included and six additional papers were selected through references in other papers. Of these 16 included articles, seven studies were related to CBD treatment for schizophrenia and eight studies described the treatment of SUD with CBD-containing compounds. Only one study assessed the effects of the treatment with medicinal cannabis for patients with a psychotic disorder and a comorbid cannabis use disorder.

### 3.1. CBD—Psychosis

Four randomized controlled trials (RCTs) and three case reports assessed the efficacy of CBD as a treatment for psychotic disorders (Table 1). 

Zuardi et al. (1995) described a 19-year-old woman with schizophrenia who received progressive increase of CBD monotherapy for 26 days (maximum of 1500 mg/day) [51]. CBD treatment was associated with the improvement of symptomatology as measured with the Brief Psychiatric Rating Scale (BPRS). This improvement did not further increase on haloperidol treatment [51]. In a second case report of the same group, three treatment-resistant schizophrenia male patients were treated with CBD monotherapy for four weeks. The authors reported mild improvement of positive and negative symptoms of one patient after CBD treatment (BPRS score decreased from 29 to 22). Moreover, CBD was well tolerated and no side effects were reported [52]. Makiol and Klunge (2019 described a case of a 57-year-old woman with treatment-resistant schizophrenia, which persisted for 21 years [53]. On admission she had a total PANSS (Positive and Negative Syndrome Scale) score of 117 and a negative symptom score of 41. Adjunctive to treatment with clozapine (275 mg/day) and lamotrigine (225 mg/day), the patient received CBD 500 mg twice daily, which was increased to 750 mg twice daily after seven weeks. On discharge, the PANSS total score decreased to 68 and negative symptom score to 21, which the authors indicated as accomplishment of remission criteria with only mild negative symptoms. CBD did not affect clozapine levels and was well tolerated apart from a mild hand tremor [53]. Leweke et al. (2012) performed a double-blind randomized controlled trial in which 39 acutely psychotic inpatients were treated with either CBD (*N =* 20) or amisulpride 800 mg (*N =* 19) for four weeks [54]. The authors did not provide information about illness duration before hospitalization. Both treatments were associated with clinical improvement, considering a decrease of positive and negative symptoms (change from baseline to 28-day assessment in positive PANSS score −9.0 ± 6.1 and −8.4 ± 7.5, and in negative PANSS score −9.1 ± 4.9 and −6.4 ± 6.0 after CBD and amisulpride, respectively; all comparisons *p* < 0.001). However, CBD treatment had a superior side-effect profile in terms of less severe changes in weight gain, extrapyramidal symptoms, prolactin levels, and sexual functioning. In addition, Leweke et al. (2012) measured anandamide levels in serum before and after treatment with CBD or amisulpride and the relationship with psychotic symptoms [54]. As compared to treatment with amisulpride, CBD showed a significant increase in anandamide levels and this was associated with the improvement of psychotic symptoms (i.e., decrease of total PANSS score). These findings suggest that anandamide levels could serve as a possible biomarker for the efficacy of CBD treatment [54]. In the largest randomized placebo-controlled trial to date, McGuire et al. (2018) assessed the effect of six-week treatment with CBD (1000 mg/day) added to antipsychotic medication in 88 moderately ill (total PANSS score >60) outpatients with schizophrenia [55]. After six weeks, positive symptoms (change from baseline −3.2 after CBD and −1.7 after placebo, treatment difference = −1.4, 95% CI = −2.5, −0.2) and global clinical impression significantly improved in the CBD group compared with placebo (treatment difference = −0.5, 95% CI = −0.8, −0.1 for improvement rates and = −0.3, 95% CI = −0.5, 0.0 for change in severity of illness) [55]. These case studies and RCTs suggest that CBD treatment for psychosis is beneficial and could possibly be as effective as antipsychotic medication. 

Two RCTs showed less conclusive results of CBD treatment on positive, negative, and cognitive symptomatology. The randomized placebo-controlled trial by Hallak et al. (2010) presented the effect of acute treatment with single doses of CBD on selective attention as measured with the Stroop Colour and Word Test in a heterogeneous group of 28 schizophrenia patients (illness duration <5 years (*N =* 11), >5 years (*N =* 17)) [56]. These patients performed the Stroop test twice: The first time without the administration of any drug and the second time after oral administration of either placebo, 300 mg, or 600 mg CBD. After the two sessions, all groups showed improvement in cognitive performance (i.e., reduced number of errors during the Stroop test). Improvement was greater in the placebo and CBD 300 mg groups, compared with the patients who received CBD 600 mg. There was no effect of CBD treatment on both positive and negative symptoms [56]. Second, the most recent double-blind, randomized placebo-controlled trial by Boggs et al. (2018) examined treatment with oral CBD (600 mg/day) or placebo adjunctive to a stable dose of antipsychotic medication in 36 chronic schizophrenia patients (mean illness duration >25 years) [57]. Although positive, negative, general, and total PANSS decreased and cognitive performance increased over time in both groups, there were no significant differences between groups. Thus, in this trial, symptomatology and cognitive performance did not improve after adjunctive CBD treatment in schizophrenia outpatients who were receiving long-term polypharmacy for a myriad of psychiatric symptoms (Boggs et al., 2018) [57]. One possibility is that these results may be explained by the significant difference in the use of multiple antipsychotics between the placebo (38.9%) and CBD groups (11%) [57].

In summary, most of abovementioned studies provided evidence for the potential of CBD as an antipsychotic treatment, which could alleviate both cognitive and psychotic symptoms in patients with psychotic disorders. The studies that showed negative results provided either a single dose of CBD [56] or included chronic schizophrenia patients who received multiple types of antipsychotic medication [57].

### 3.2. CBD—Substance Use Disorders

To date, eight studies assessed the potential effect of CBD as treatment for SUD (Table 2). Six studies focussed on cannabis dependence and two on tobacco dependence. 

#### 3.2.1. Cannabis Dependence 

The treatment of cannabis dependence with a cannabis-extracted CBD/THC mixture (Sativex) was assessed in three clinical trials. In the first double-blind randomized controlled trial by Allsop et al. (2014), 51 inpatients with cannabis dependence received Sativex or placebo for six days along with cognitive behavioural therapy [58]. Immediately after treatment, Sativex significantly decreased cannabis withdrawal and craving symptoms and improved treatment retention rates. At 28 days, both groups showed a decrease in cannabis use, in the amount of cannabis-related problems, and in the severity of cannabis dependence from baseline to follow-up, but the differences between groups were no longer significant [58]. A second double-blind randomized placebo-controlled trial assessed the effects of an eight-week treatment with self-titrated or fixed doses of Sativex in nine subjects with cannabis dependence [59]. During treatment sessions, when cannabis use was not allowed, both fixed and self-titrated doses of Sativex reduced cannabis withdrawal symptoms, however, the high fixed dose seemed the most effective. Sativex did not influence cannabis craving. The same research group performed a larger double-blind randomized placebo-controlled trial in which 27 cannabis-dependent subjects were treated with self-titrated dosages of Sativex in combination with cognitive behavioural therapy over 12 weeks [60]. The abstinence rate did not change significantly between baseline and follow-up. Cannabis use, withdrawal, and craving symptoms reduced over time in both groups. Sativex was associated with a greater reduction in cannabis craving symptoms when compared with placebo.

While the previous studies used CBD/THC mixtures, the following three studies described treatments of cannabis dependence with pure CBD. The first study by Crippa et al. (2013), reported a 19-year-old female with cannabis dependence who was treated with oral CBD over 11 days [61]. The dose was 300 mg on day 1, 600 mg on days 2–10 and 300 mg on day 11. During treatment, cannabis withdrawal, anxiety, and dissociative symptoms progressively decreased. A six-month follow-up period revealed a relapse of cannabis use, however at a lower frequency than on admission [61]. In a second case report, Shannon and Opila-Lehman (2015) described the treatment with 24–18 mg CBD oil adjunctive to citalopram and lamotrigine for a 27-year-old male with cannabis disorder and bipolar disorder. During the use of CBD oil, the patient did not use cannabis, showed a decrease in anxiety, and demonstrated improved sleep quality [62]. Third, the open-label clinical trial by Solowij et al. (2018) assessed the effects of ten-week treatment with CBD (200 mg/day) on psychological symptoms, cognition, and plasma concentrations [63]. Twenty frequent and ongoing cannabis users, of which twelve were dependent users (severity dependence scale score ≥3) and ten were nondependent users (severity dependence scale score <3), participated in this trial. Between baseline and post treatment sessions, cannabis use and withdrawal did not change, but cannabis-related experiences (i.e., euphoria and feeling high) decreased. Anxiety, depressive, and psychotic-like symptoms showed greater reductions in dependent than nondependent users. Attentional switching, verbal learning, and memory improved in all participants. Remarkably, higher CBD plasma concentrations were associated with lower psychotic-like symptoms (total and negative), distress, anxiety, and severity of cannabis dependence [63]. These results suggest greater effects of CBD in dependent users which can possibly be detected through CBD plasma concentrations. 

Taken collectively, CBD shows some promise in the treatment of cannabis dependence as it reduces behaviour relevant to addiction such as craving and withdrawal in almost all studies. Because double-blind placebo-controlled RCTs with pure CBD are lacking, the evidence for the efficacy of products containing a combination of CBD and THC in the treatment of cannabis dependence is more convincing. 

#### 3.2.2. Tobacco Dependence

Morgan et al. (2013) assessed the effects of the optional use of an inhaler containing CBD (400 µg/dose) during one week in 24 individuals who smoked >10 cigarettes/day and intended to quit [64]. Results showed that CBD reduced the total number of cigarettes smoked during the treatment period. However, CBD did not have an effect on craving symptoms. In addition, craving was reduced in both groups at the end of treatment, but this did not maintain at follow-up [64]. Additionally, a second clinical trial into the efficacy of CBD treatment for tobacco dependence provided information about treatment outcomes related to motivation and evaluation. Hindocha et al. (2018) treated 30 tobacco- dependent individuals with a single dose of CBD 800 mg [65]. Attentional bias to pictorial cigarette cues was measured using a visual probe and an explicit rating task. In addition, craving, withdrawal, and side effects were assessed. After overnight cigarette abstinence, CBD reduced attentional bias to cigarette cues and pleasantness of cigarette cues, which could suggest that CBD has a potential effect on the motivational aspects of addiction. In this trial, CBD did not have an effect on craving and withdrawal. Moreover, no significant differences were found between CBD and placebo on side effects [65]. 

### 3.3. CBD—Psychosis and SUD

Schipper and colleagues (2018) were the first who described the efficacy of CBD treatment for patients with a psychotic disorder and a comorbid treatment-resistant cannabis use disorder (Table 3) [66]. Seven hospitalized patients received eight weeks of treatment with Bedrolite, medicinal cannabis that contains 0.4% THC and 9% CBD, as add-on therapy to conventional antipsychotic medication. The medicinal cannabis was supposed to substitute street cannabis used by the patients but was only provided at fixed moments during the day. Doses ranged from 0.125 to 0.5 g daily (11–45 mg CBD), depending on dose and frequency of the use of street cannabis before admission. Treatment with CBD-rich medicinal cannabis did not affect psychosis- or dependence-related symptomatology. Patients preferred street cannabis over the medicinal cannabis and started to use additional street cannabis during the treatment program [66]. The most likely explanation for these negative results was the low THC concentration in Bedrolite as compared to street cannabis. As a result, the substitution of THC-rich street cannabis by medicinal cannabis with mainly CBD may have been too abrupt for most patients. 

## 4. Discussion and Conclusions

The current review aimed to provide a detailed and up-to-date systematic literature overview of studies that investigated the efficacy of CBD treatment for schizophrenia and/or SUD. Based on this overview, a second aim was to examine whether there is a specific subgroup of patients with schizophrenia, SUD, or both that may benefit most from CBD treatment. In some but not all studies, CBD seemed effective as a treatment for psychosis and SUD. CBD may have the capacity to alleviate positive, negative, and cognitive symptoms in schizophrenia, as well as craving and withdrawal in SUD. Although most of the studies showed promising results, differences in study design, patient population, and use of concomitant medication make it difficult to define specific subgroups to whom CBD should be administered. In addition, CBD doses and administration were different between studies and most of the reviewed studies did not describe the source of CBD (i.e., synthetic or cannabis extracted), which may have different efficacy. However, the results of the reviewed studies suggested some features that may contribute to the identification of patients who may benefit most from CBD treatment.

Research into CBD treatment for psychosis provided evidence for a few possible clinical and biological characteristics of the subgroup. The effects of CBD were studied in patients in both early and later stages of psychotic disorders. Overall, acutely psychotic and early onset patients demonstrated reductions of positive and negative symptoms [51,54], while treatment resistant and chronic patients showed less promising improvement [56,57]. Even though Makiol and Kluge (2019) described a chronic schizophrenia patient who exhibited great clinical improvement (i.e., change of total PANSS score: 49) [53], the majority of the results suggest that CBD may be more effective in the early stage of psychotic disorders. This is in line with previous studies suggesting that immune dysregulation (i.e., microglial activation) is mainly involved in the early stage of psychotic disorders [67,68]. As cannabinoid receptors are also present on microglia, it is possible that CBD exerts its effects by decreasing microglial activity [69]. Furthermore, anandamide levels in serum could serve as a possible biomarker for the efficacy of CBD treatment. For instance, Leweke et al. (2012) reported a significant increase in anandamide levels after CBD treatment, which was associated with the improvement of psychotic symptoms (i.e., decrease of total PANSS score) [54]. This is in concurrence with a previously reported inverse association between elevated anandamide levels in cerebrospinal fluid and psychotic symptoms in antipsychotic-naïve patients [29,30,31].

Research into CBD treatment for SUD primarily focussed on cannabis dependence. Taken collectively, CBD shows promise in the treatment of cannabis dependence as it reduces craving and withdrawal in almost all studies. However, these studies have heterogeneous study designs and administration methods. The differences in administration and dosages may provide a possible explanation for the different results observed in the included studies. For instance, THC/CBD mixtures might be more effective in reducing some features of cannabis dependence (i.e., craving, use and withdrawal) than pure CBD. Moreover, the level of cannabis dependence and intrinsic motivation for treatment, may help to define a possible subgroup of patients in which CBD is more effective. Dependent users (i.e., those with a severity dependence scale score ≥3), showed reduced anxiety, depression, and psychotic-like symptoms after a 10-week treatment with CBD, compared with nondependent users [63]. However, studies that include individuals with more symptoms at baseline can show greater reductions after treatment. Therefore, it is difficult to determine whether symptom severity is truly a patient characteristic that could predict better outcomes after CBD treatment. Solowij et al. (2018) also found that cannabis-related experiences decreased after treatment [63], which is in accordance with previous studies that indicate that CBD counteracts the effects induced by THC [46,47]. Intrinsic motivation for treatment seems an important aspect as well, as it may increase medication adherence [65]. Conversely, patients that do not seek treatment are less inclined to follow strict study protocols [66]. The majority of the discussed studies recruited individuals with cannabis dependence from the community, which suggests that these individuals were at least open for treatment. To a certain extent, this may explain why the study by Schipper et al. (2018) found that CBD administration was not effective [66], as they included individuals that did not seek treatment.

Considering the efficacy of CBD in both psychotic disorders and SUD, one can speculate that CBD should also be effective in the treatment of the comorbidity. However, only Schipper et al. (2018) studied this population, with negative results [66]. As discussed previously, these patients were treatment resistant for SUD and showed lack of motivation for treatment. An additional limitation of this study was the good baseline functioning in five out of seven patients. Moreover, this study administered CBD in a formulation that contained very little THC, which possibly explains why the participants preferred street cannabis.

Future studies could take these limitations into account and should focus on examining the effects of CBD in the different stages of psychotic disorders, considering the high prevalence of comorbid SUD. Studies into psychotic disorders could use CBD (i.e., either as monotherapy or add-on) to treat psychotic symptoms and to prevent relapse in early stages, while exploring the effects on comorbid substance use (e.g., cannabis). These studies should use standardized measures to assess cannabis use. In later stages and comorbid treatment-resistant SUD, CBD studies may aim to reduce cannabis use, using harm-reduction strategies (e.g., gradually shift the THC/CBD ratio in medicinal cannabis in favour of CBD) [70]. Currently, nine ongoing clinical trials that study the effects of CBD on psychotic disorders or SUD (including alcohol and cocaine misuse) are registered in clinicaltrials.gov, of which one (NCT03883360) includes patients with recent-onset psychotic disorder and cannabis use. Therefore, more results on this topic are expected in the near future.

It remains unclear if the efficacy of CBD in schizophrenia, addiction, and their comorbidity could be explained by shared or different biological mechanisms. To elucidate this, future efforts should be taken to study the relationship between the eCB system, GABA/glutamate, and the immune system. For example, neuroimaging studies (e.g., positron-emission tomography, PET and magnetic resonance spectroscopy, MRS) could measure CB1 receptor densities and markers for glia in patients with schizophrenia and/or SUD who were treated with CBD.

In conclusion, CBD treatment is a promising and novel tool with several potential applications in the treatment of psychotic disorders, substance use disorders, and their comorbidity. Large-scale trials are needed to establish its clinical utility.

## Figures and Tables

**Table 1:** Case reports and clinical trials on the efficacy of cannabidiol (CBD) as a treatment for psychotic disorders.

Study	Study Design	Participants	Substance Use	Intervention	CBD Administration	Primary Outcomes
Zuardi et al. (1995) [51]	Case report	19-year-old female schizophrenia inpatient (two years after first hospitalization)	Not reported	Progressive increase of CBD monotherapy over four weeks, followed by haloperidol treatment	Oral; up to 1500 mg/day	Improvement of symptomatology. Improvement did not continue on haloperidol.No side effects.
Zuardi et al. (2006) [52]	Case series	Three male inpatients with treatment-resistant schizophrenia	Not reported	Progressive increase of CBD monotherapy over four weeks, followed by olanzapine treatment	Oral; up to 1280 mg/day	Mild improvement of symptomatology of one patient after CBD treatment.No side effects.
Makiol and Klunge (2019) [53]	Case report	57-year old-female treatment-resistant schizophrenia inpatient	Not reported	Treatment with CBD adjunctive to clozapine and lamotrigine	Oral; up to 1500 mg/day	Improvement of symptomatology and the patient fulfilled remission criteria with only mild negative symptoms.
Leweke et al. (2012) [54]	Double-blind CBD vs. amisulpride RCT	39 acutely psychotic inpatients	Not reported, exclusion criteria were SUD or positive urine drug screening for illicit drugs in general and cannabis in particular.	Hospitalization and four-week treatment with CBD or amisulpride	Oral; up to 800 mg/day	Treatment with either CBD or amisulpride is associated with improvement of symptomatology, but CBD has a superior side-effect profile.
McGuire et al. (2018) [55]	Double-blind placebo RCT	88 outpatients with schizophrenia	Not reported, substance use was not an exclusion and not prohibited during the study.	A six-week treatment with CBD adjunctive to antipsychotic medication.	Oral solution; 1000 mg/day	Improvement of symptomatology, no side effects.
Hallak et al. (2010) [56]	Single dose double-blind placebo RCT	28 schizophrenia outpatients	Not reported	Acute treatment with a single dose of CBD	Oral; 300 or 600 mg	CBD 300 mg and placebo both improved cognitive performance as compared to CBD 600 mg. No effects on symptomatology.
Boggs et al. (2018) [57]	Double-blind placebo RCT	36 outpatients with chronic schizophrenia	Not reported, patients with substance abuse in the past three months or dependence in the past six months were excluded.	Six-week treatment with CBD added to a stable dose of antipsychotic medication	Oral; 600 mg/day	Cognitive performance improved after placebo, symptomatology improved in both groups, no differences between groups.

CBD: Cannabidiol; RCT: Randomized clinical trial.

**Table 2:** Case reports and clinical trials on the efficacy of CBD as a treatment for substance use disorders.

Study	Study Design	Participants	Intervention	CBD Administration	Primary Outcomes
**Cannabis Dependence**					
Allsop et al. (2014) [58]	Double-blind placebo RCT	51 inpatients with cannabis dependence	A six-day treatment with Sativex in combination with CBT	Intranasal; maximum daily: 86.4 mg THC and 80 mg CBD	Sativex reduced cannabis withdrawal and cravings, and improved treatment retention rates.
Trigo et al. (2016) [59]	Double-blind placebo RCT	Nine individuals with cannabis dependence	Eight-week treatment with self-titrated or fixed doses of Sativex or placebo.	Intranasal; up to 108 mg THC and 100 mg CBD	During interruption of cannabis use both fixed and titrated doses of Sativex reduced cannabis withdrawal symptoms (but not craving), however the high fixed dose seemed the most effective.
Trigo et al. (2018) [60]	Double-blind placebo RCT	27 individuals with cannabis dependence	Twelve-week treatment with self-titrated dosages of Sativex next to weekly CBT sessions.	Intranasal; up to 113.4 mg THC and 105 mg CBD/day	Cannabis use, cravings, and withdrawal decreased in both groups over time. Sativex reduced cannabis cravings.
Crippa et al. (2013) [61]	Case report	19-year-old female diagnosed with cannabis dependence	Hospitalization and progressive increase of CBD	Oral; 300 to 600 mg	A progressive reduction of cannabis withdrawal, anxiety, and dissociative symptoms.
Shannon and Opila-Lehman (2015) [62]	Case report	27-year-old male diagnosed with bipolar disorder and cannabis dependence	Treatment with CBD oil added to citalopram and lamotrigine	Intranasal; decreasing from 24 to 18 mg	Abstinence from cannabis, better sleep quality, and decrease in anxiety during the use of CBD oil.
Solowij et al. (2018) [63]	Open-label clinical trial	20 ongoing cannabis users	Ten-week treatment with CBD	Oral; 200 mg daily	CBD improved psychological and cognitive symptomatology. Greater benefits were observed in dependent than in nondependent cannabis users.
**Tobacco Dependence**					
Morgan et al. (2013) [64]	Double-blind placebo RCT	24 individuals who smoked >10 cigarettes per day and intended to quit	Optional CBD treatment during one week	Inhalation; 400 µg CBD per dose	CBD reduced the total number of cigarettes smoked. Reduction of craving in both groups after one week of treatment, but this did not maintain at follow-up.
Hindocha et al. (2018) [65]	Single dose double-blind placebo RCT	30 individuals with tobacco dependence	Treatment with a single dose of CBD after an overnight of cigarette abstinence	Oral; 800 mg CBD	CBD reduced the salience and peasantness of cigarette cues but had no effect on craving and withdrawal.

CBD: Cannabidiol; CBT: Cognitive behavioural therapy; RCT: Randomized clinical trial; THC: Δ9-Tetrahydrocannabinol.

**Table 3:** Studies on CBD treatment for patients with a psychotic disorder and a comorbid cannabis use disorder.

Study	Study Design	Participants	Intervention	CBD Administration	Primary Outcomes
Schipper et al. (2018) [66]	Case report	Seven inpatients with a psychotic disorder and a comorbid treatment-resistant cannabis use disorder	Eight-week treatment with medicinal cannabis (Bedrolite: 0.4% THC and 9% CBD) adjunctive to antipsychotic medication.	Inhalation: 11–45 mg/day	No effect on symptomatology or craving.

CBD: Cannabidiol; THC: Δ 9-tetrahydrocannabinol.
